# PMF-seq: a highly scalable screening strategy for linking genetics to mitochondrial bioenergetics

**DOI:** 10.1038/s42255-024-00994-0

**Published:** 2024-02-27

**Authors:** Tsz-Leung To, Jason G. McCoy, Naomi K. Ostriker, Lev S. Sandler, Carmen A. Mannella, Vamsi K. Mootha

**Affiliations:** 1 Howard Hughes Medical Institute and Department of Molecular Biology, Massachusetts General Hospital, Boston, MA USA; 2 Broad Institute of MIT and Harvard, Cambridge, MA USA; 3 Department of Systems Biology, Harvard Medical School, Boston, MA USA; 4 Department of Physiology, Center for Biomedical Engineering and Technology, University of Maryland School of Medicine, Baltimore, MD USA

**Keywords:** High-throughput screening, Genetics research, Metabolism, Bioenergetics, Mitochondria

## Abstract

Our current understanding of mitochondrial organelle physiology has benefited from two broad approaches: classically, cuvette-based measurements with suspensions of isolated mitochondria, in which bioenergetic parameters are monitored acutely in response to respiratory chain substrates and inhibitors^1–4^, and more recently, highly scalable genetic screens for fitness phenotypes associated with coarse-grained properties of the mitochondrial state^5–10^. Here we introduce permeabilized-cell mitochondrial function sequencing (PMF-seq) to combine strengths of these two approaches to connect genes to detailed bioenergetic phenotypes. In PMF-seq, the plasma membranes within a pool of CRISPR mutagenized cells are gently permeabilized under conditions that preserve mitochondrial physiology, where detailed bioenergetics can be probed in the same way as with isolated organelles. Cells with desired bioenergetic parameters are selected optically using flow cytometry and subjected to next-generation sequencing. Using PMF-seq, we recover genes differentially required for mitochondrial respiratory chain branching and reversibility. We demonstrate that human d-lactate dehydrogenase specifically conveys electrons from d-lactate into cytochrome *c* to support mitochondrial membrane polarization. Finally, we screen for genetic modifiers of tBID, a pro-apoptotic protein that acts directly and acutely on mitochondria. We find the loss of the complex V assembly factor ATPAF2 acts as a genetic sensitizer of tBID’s acute action. We anticipate that PMF-seq will be valuable for defining genes critical to the physiology of mitochondria and other organelles.

## Abstract (web summary)

The authors report a genetic screening method that preserves mitochondrial physiology under cell permeabilization, which allows in-depth genetic dissection of mitochondrial bioenergetics.

## Main

**Fig. 1:**
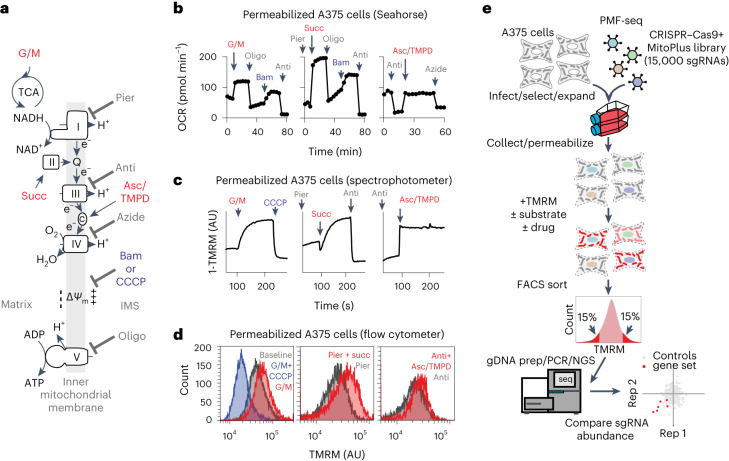
Schematic overview of PMF-seq. **a**, Schematic diagram of how substrates can feed into the respiratory chain to support the membrane potential. **b**, OCR of permeabilized A375 cells as measured by Seahorse analyser with glutamate/malate (left), succinate (middle) or ascorbate/TMPD (right) as the substrate. **c**, Kinetics of Δ*Ψ*_m_ measured as TMRM fluorescence in permeabilized A375 cells with glutamate/malate (left), succinate (middle) or ascorbate/TMPD (right) as the substrate. **d**, Endpoint Δ*Ψ*_m_ of permeabilized A375 cells measured as TMRM fluorescence by a flow cytometer with glutamate/malate (left), succinate (middle) or ascorbate/TMPD (right) as the substrate. **e**, Experimental workflow of PMF-seq in permeabilized A375 cells. IMS, intermembrane space; G/M, glutamate/malate; Succ, succinate; Pier, piericidin A; Anti, antimycin A; Oligo, oligomycin A; Bam, BAM15; CCCP, carbonyl cyanide *m*-chlorophenyl hydrazone; AU, arbitrary units. Source data

Early insights into coupling mechanisms in oxidative phosphorylation (OXPHOS) came from studies on suspensions of isolated mitochondria, and this allows monitoring of key parameters such as oxygen consumption, membrane potential, NADH oxidation and calcium uptake following titration of substrates and inhibitors that are not cell permeable^1–4^. When reducing equivalents are fed to any of the respiratory chain complexes I–IV (Fig. 1a), energy is conserved by coupling electron transport to the generation of a proton motive force across the inner membrane, consisting of a mitochondrial membrane potential (Δ*Ψ*_m_) and a pH gradient (ΔpH). This leads to a stable, high membrane potential state (state 4), which can be rapidly dissipated by the F_1_F_o_-ATPase complex V following the addition of adenosine diphosphate (ADP, state 3). For example, mitochondria can be energized with glutamate/malate that feeds specifically into complex I or succinate/piericidin, a substrate/inhibitor pair that feeds directly into complex II (Fig. 1a). These classical approaches made it possible to investigate the mitochondrial respiratory chain and the ordering of its complexes using highly defined media conditions, albeit at low throughput.

A subsequent and highly complementary approach for investigating mitochondrial biology is genetic screening. For example, studies on yeast genetics were crucial in delineating pathways required for the function and assembly of cytochrome *c* oxidase^5^. Additionally, genetic analysis of cell lines from patients with inherited mitochondrial disease has led to the discovery of numerous respiratory chain assembly factors—notably those of complex I (ref. ^6^), which is missing in the genetically tractable yeast *Saccharomyces cerevisiae*^7^. Recently, pooled clustered regularly interspaced short palindromic repeats (CRISPR) genetic screening has made it possible to systematically identify genes required for OXPHOS on the basis of cellular growth phenotypes that are dependent on intact mitochondrial function^8–10^. However, these genetic studies are inherently coarse grained and cannot provide fine resolution on detailed organelle bioenergetics.

Ideally, we would combine the power of classical bioenergetics—in which substrates and inhibitors can be carefully fed to the mitochondrial respiratory chain—with highly parallel genetic screening that employs modern technologies such as CRISPR. Such hybrid technology would ideally be scalable so that we could systematically connect genes to mitochondrial physiology with the biochemical resolution afforded by classical studies of bioenergetics in isolated mitochondria.

In this Letter, we report the development of such a technology, which we call permeabilized-cell mitochondrial function sequencing (PMF-seq). The core concept behind our approach is to interrogate mitochondrial function in mutagenized cell pools where the plasma membrane has been permeabilized but the mitochondria have been left physiologically competent and accessible to substrates/inhibitors. We reasoned that by CRISPR mutagenizing cultured cells before permeabilization we could perform kinetic measurements in bulk, sort cells based on a bioenergetic parameter at a specified time and perform next-generation sequencing, thereby connecting genes with bioenergetics.

We began by optimizing cell permeabilization and buffer conditions so that mitochondria remained functionally intact and single cells could still be reliably sorted. We used perfringolysin O (ref. ^11^), a bacterial toxin that selectively permeabilizes cholesterol-rich membranes, such as the plasma membrane, by forming giant ring-shaped pores. We confirmed plasma membrane permeabilization and substrate-specific respiration in A375 cells using plasma membrane-impermeable glutamate/malate (complex I), succinate (complex II) and ascorbate/*N*,*N*,*N*′,*N*′-tetramethyl-*p*-phenylenediamine (TMPD) (cytochrome *c*) with the Seahorse XFe96 analyser (Fig. 1b). We could monitor both the basal steady state and energized steady states of Δ*Ψ*_m_ in pools of permeabilized cells using the lipophilic cationic dye tetramethylrhodamine methyl ester (TMRM; Fig. 1c). We then repeated the same experiment and could detect low and high Δ*Ψ*_m_ states as an endpoint assay on a flow cytometer (Fig. 1d). These studies show that, in suspensions of permeabilized cells, mitochondria can be energized using specific substrate/inhibitor combinations that are otherwise cell impermeable. They further show that the combination of flow cytometry and TMRM as a Δ*Ψ*_m_ probe enables reliable detection of steady-state endpoints from kinetic measurements.

For the PMF-seq workflow, we combined high-throughput pooled CRISPR–Cas9 mutagenesis with a TMRM readout by fluorescence-activated cell sorting (FACS; Fig. 1e). We generated a custom CRISPR single guide RNA (sgRNA) library targeting genes encoding the mitochondrial proteome^12^ and the lysosomal proteome^13^ and genes from key metabolic pathways, such as glycolysis. The resulting CRISPR library, which we term ‘MitoPlus’, consists of 15,271 sgRNAs targeting 1,864 genes. We lentivirally infected wild-type A375 cells with CRISPR–Cas9 and the MitoPlus library. CRISPR-mutagenized cells were collected on day 15 post-infection, permeabilized and stained with TMRM, treated with specific substrate/inhibitor combinations and subjected to FACS. The same forward-versus-side-scatter gating was applied to the cell population at all timepoints after plasma membrane permeabilization to filter out debris, dead cells, multiplets and exceptionally large cells (Supplementary Information). This gated population remained stable over the course of the sorting process (~15 min). Cells corresponding to the top and bottom ~15% of the TMRM distributions (designated as ‘high tail’ and ‘low tail’) were collected, with genomic DNA collected and subjected to polymerase chain reaction (PCR) amplification and guide RNA sequencing. Analyses of the screening results were performed using a *Z-*score-based method as previously described^9^. In this analysis, genes required for generating the membrane potential have highly negative *Z-*scores (Supplementary Information).

We applied PMF-seq to uncover the known genetic basis of respiratory chain branching and reversibility, which cannot be readily probed in intact cell screens. Although the respiratory chain is traditionally depicted as a simple linear sequence of protein complexes, it is in fact highly branched^14^, with multiple inputs into coenzyme Q (for example, complex I, SDH, GPD2, DHODH, ETFDH and SQOR) and into cytochrome *c* (for example, complex III, MIA40 and SUOX). Nearly all OXPHOS complexes are also reversible, depending on the prevailing proton motive force and substrate concentrations. Traditional CRISPR screens in intact cells identify genes broadly required for the OXPHOS system^8^. However, they do not provide insight into genes differentially required for branching or for less commonly used substrates. In theory the advantage of PMF-seq is that the substrate and inhibitor milieu can be controlled, forcing the cell to utilize a pathway whose genetic basis can then be queried.

**Fig. 2:**
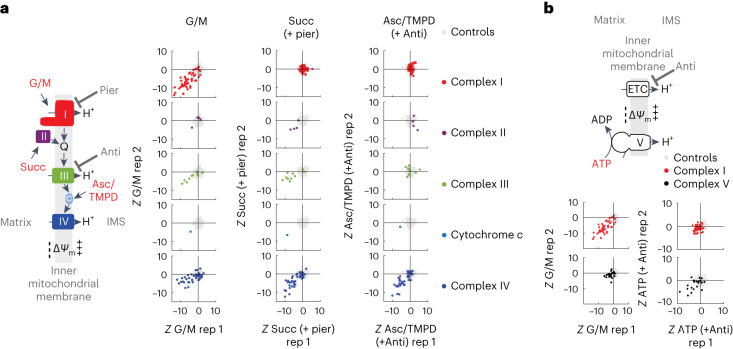
Genetic dissection of OXPHOS branching and reversibility. **a**, Scatterplots of *Z*-scores of the specified respiratory chain components when glutamate/malate (first column), succinate (second column), or ascorbate/TMPD (third column) was used as the respiratory chain substrate in permeabilized A375 cells. **b**, Scatterplots of *Z*-scores of complex I (red) or complex V (black) when glutamate/malate (first column) or ATP (second column) was used as the substrate. Data from biological duplicates are shown. A highly negative *Z*-score indicates the enrichment of sgRNAs for a given gene in the low tail of the TMRM distribution, suggesting the dependency on that gene for membrane potential generation. Source data

**Extended Data Fig. 1:**
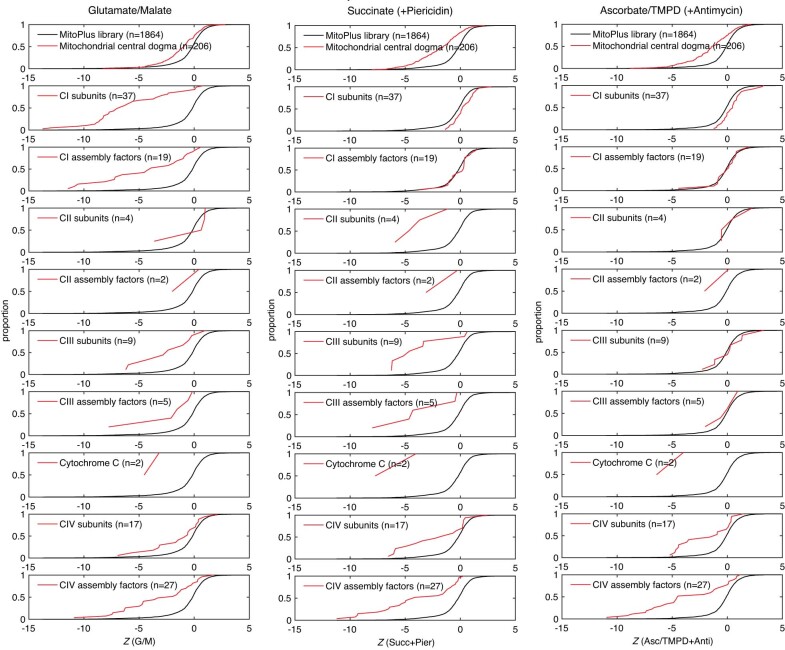
Specificity across PMF-seq screens. OXPHOS gene sets are annotated in the MitoCarta3.0 database and the cumulative distribution function of each selected gene set (red curve) is plotted against that of all genes (black curve) within the MitoPlus library in PMF-seq experiments using glutamate/malate (left column), succinate (middle column), or ascorbate/TMPD (right column) as substrates in A375 cells (Fig. 2a). Source data

**Extended Data Fig. 2:**
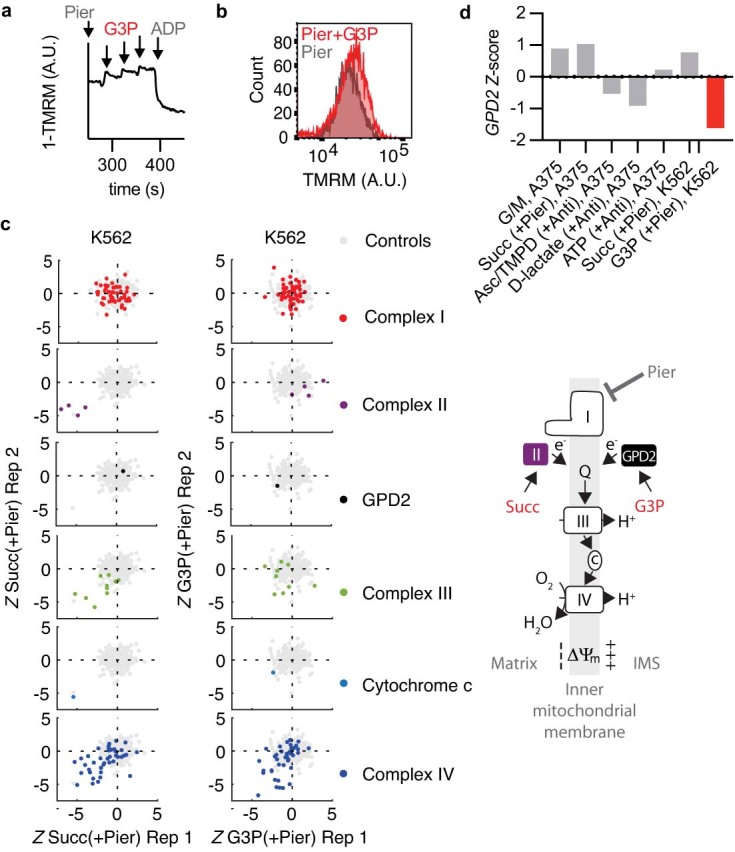
Genetic basis of branching at coenzyme Q. **a**, Kinetics of mitochondrial membrane potential measured as TMRM fluorescence in permeabilized K562 cells with glycerol 3-phosphate (G3P) as the substrate. **b**, Endpoint mitochondrial membrane potential measured as TMRM fluorescence by a flow cytometer in permeabilized K562 cells with G3P as the substrate. **c**, Scatterplots of *Z*-scores of the specified respiratory chain components when succinate (first column), or glycerol 3-phosphate (second column) was used as the respiratory chain substrate in permeabilized K562 cells. Data from biological duplicates are shown. A highly negative *Z*-score indicates the enrichment of sgRNAs for a given gene in the low tail of the TMRM distribution, suggesting the dependency on that gene for membrane potential generation. **d**. Mean *Z* scores for *GPD2* across screens. G3P, glycerol 3-phosphate; IMS, intermembrane space; G/M, glutamate/malate; Succ, succinate; Pier, piericidin A; Anti, antimycin A. Source data

**Extended Data Fig. 3:**
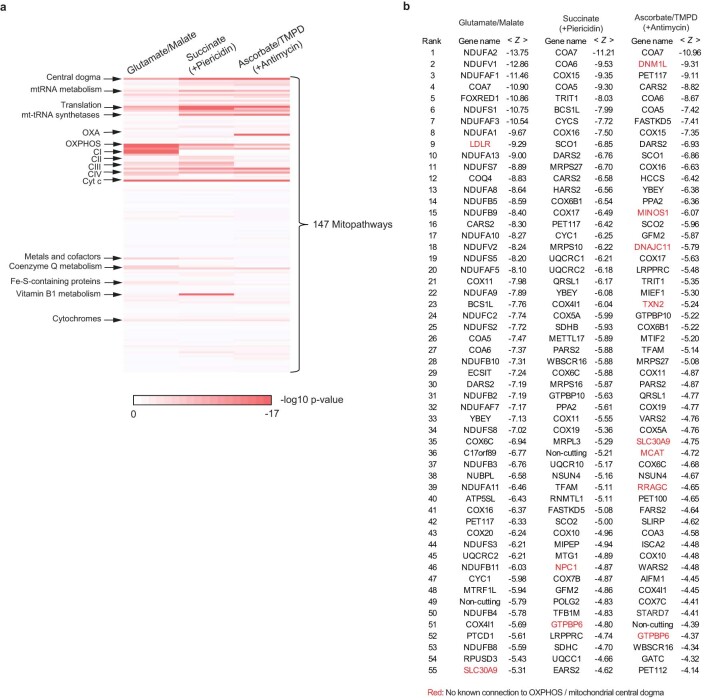
Additional data related to Fig. 2a. **a**, The 147 ‘Mitopathways’ gene sets are defined in the MitoCarta 3.0 database. The heatmap displays log base 10 p-values for each pathway, derived from overlap based on cumulative hypergeometric test statistics with a *Z*-score cutoff of −2 in PMF-seq experiments using glutamate/malate (left panel), succinate (middle panel), or ascorbate/TMPD (right panel) as substrates in A375 cells (Fig. 2a). **b**, Top hits in PMF-seq experiments as determined by the lowest mean *Z* scores from both replicates. Source data

In agreement with the theory, we find that PMF-seq recovers genes encoding the structural subunits of complex I only when glutamate/malate (a classic complex I cocktail) is used, complex II genes only under conditions of succinate/piericidin (a classic complex II cocktail), and complex III genes only when coenzyme Q-linked substrates are provided (Fig. 2a). As expected, the downstream respiratory chain complexes cytochrome *c* and complex IV score for all these substrates (Fig. 2a). Importantly, PMF-seq also identifies genes encoding the specific assembly factors for respiratory chain complexes with substrate specificity (Extended Data Fig. 1). Consistent with prior studies^15^, we found that glycerol 3-phosphate can energize mitochondria in K562 cells when complex I is inhibited by piericidin (Extended Data Fig. 2a,b). PMF-seq in K562 cells revealed a specific dependency on SDH genes (complex II) with succinate and on *GPD2* with glycerol 3-phosphate (Extended Data Fig. 2c). The dependency on *GPD2* is specific to the glycerol 3-phosphate screen (Extended Data Fig. 2d). These results demonstrate that PMF-seq is able to identify the branchpoints into the respiratory chain with specificity. As expected, PMF-seq identifies genetic requirements in areas such as mitochondrial central dogma, mtRNA metabolism, mitochondrial translation, coenzyme Q metabolism and metal cofactors (Extended Data Fig. 3a)—which are required for all components of the respiratory chain independent of substrate—as well as a small group of genes not previously associated with OXPHOS (Extended Data Fig. 3b). Genes such as *LDLR* and *NPC1* probably scored for technical reasons, namely due to their influence on cholesterol, a key component in perfringolysin O-mediated plasma membrane permeabilization.

**Extended Data Fig. 4:**
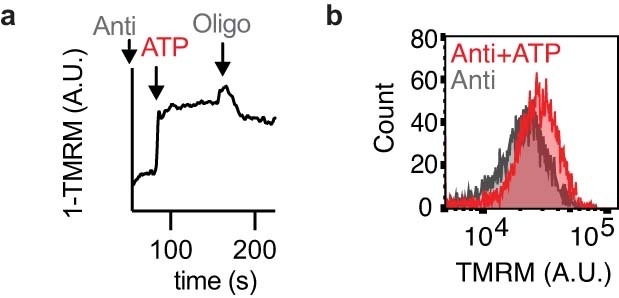
Additional data related to Fig. 2b. **a**, Kinetics of mitochondrial membrane potential measured as TMRM fluorescence in permeabilized A375 cells with ATP as the substrate. **b**, Endpoint mitochondrial membrane potential measured as TMRM fluorescence by a flow cytometer in permeabilized A375 cells with ATP as the substrate. Anti: antimycin A; Oligo: oligomycin A. Source data

We probed the genetic basis for complex V reversal, whereby glycolytic ATP is consumed to support Δ*Ψ*_m_ (ref. ^16^). We confirmed in our permeabilized A375 system that added ATP can energize mitochondria when the respiratory chain is inhibited at complex III by antimycin (Extended Data Fig. 4a), and we further showed that ATP can support the membrane potential during FACS sorting (Extended Data Fig. 4b). PMF-seq reveals strong genetic dependency on known complex V genes when ATP, but not other substrates, is provided (Fig. 2b). Surprisingly, we also observed a strong dependency on complex III and complex IV in the ATP PMF-seq experiment, indicating that these two complexes may be required for reverse action of complex V. This result is non-intuitive and merits future follow-up.

We next used PMF-seq to provide genetic insight into d-lactate as a respiratory fuel. In *S. cerevisiae* and *Arabidopsis thaliana*, d-lactate can feed into the respiratory chain via the enzyme d-lactate dehydrogenase (LDHD), which transfers electrons directly to cytochrome *c* by oxidizing d-lactate^17,18^. A recent study demonstrated that in human T cells, d-lactate activates the mitochondrial electron transport chain to promote ATP production, though this work reported a distinct, LDHD-independent mechanism^19^. Human *LDHD* deficiency has been linked to diverse pathologies—ranging from d-lactic acidosis, a complication of short bowel syndrome^20^, to complex IV deficiency with neurological manifestations^21^. While human LDHD is known to be associated with the mitochondrial inner membrane^22^, to the best of our knowledge, its role in electron transfer from d-lactate to cytochrome *c* has never been directly demonstrated. Rather, previous work on human LDHD has been focused on detoxification of reactive metabolites.

**Fig. 3:**
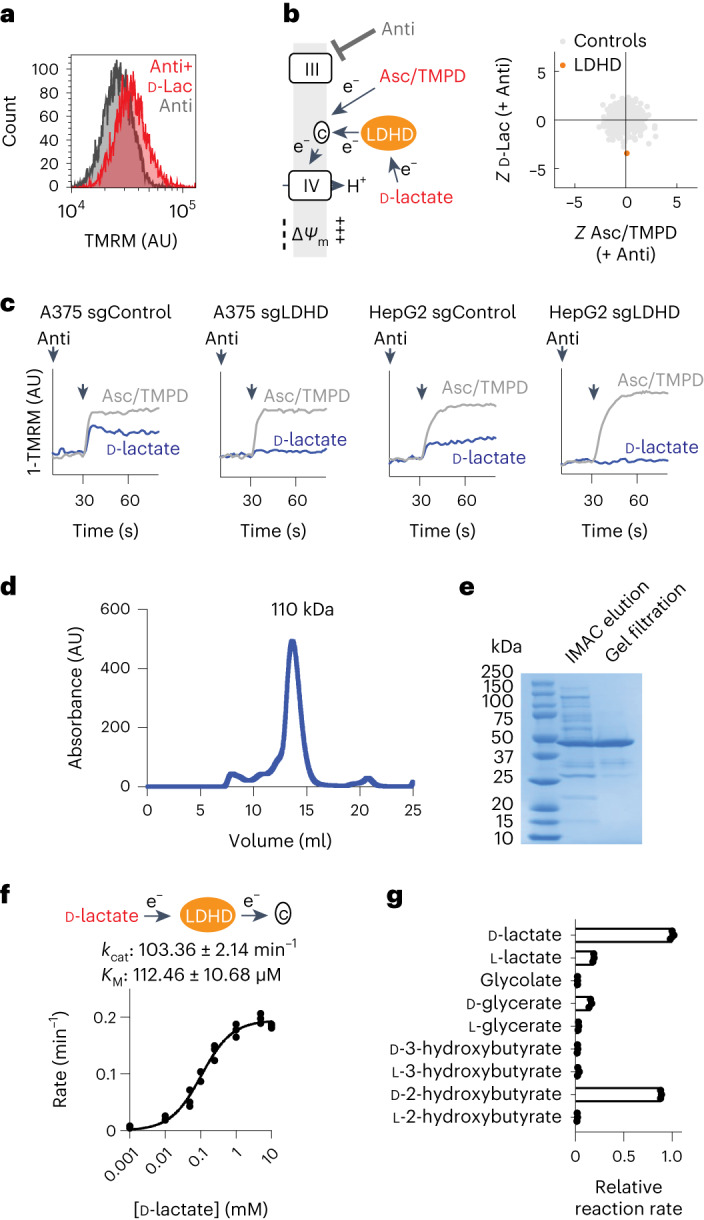
PMF-seq reveals LDHD is required for utilization of d-lactate as a respiratory chain fuel in human cells. **a**, Endpoint Δ*Ψ*_m_ measured as TMRM fluorescence by a flow cytometer for permeabilized A375 cells with d-lactate as the substrate. **b**, Scatterplot of *Z*-scores of LDHD when ascorbate/TMPD or d-lactate was used as the substrate. **c**, Kinetics of Δ*Ψ*_m_, measured as TMRM fluorescence, in permeabilized control and *LDHD* KO cells for A375 (left and middle left) and HepG2 (middle right and right). Ascorbate/TMPD (grey line) or d-lactate (blue line) was added as the substrate after antimycin A treatment, as indicated by the second arrow. **d**, Size exclusion chromatography profile of purified human LDHD. **e**, SDS–PAGE analysis of purified human LDHD visualized with Coomassie stains. **f**, Reaction scheme and in vitro steady-state enzyme kinetics of LDHD-catalysed cytochrome *c* reduction by d-lactate. **g**, Relative initial reaction rates of LDHD-catalysed cytochrome *c* reduction by 10 mM of the specified substrates. Shown in **e** is a representative gel image from a purification that has been performed five times. Shown are *n* = 3 individual replicates in **f** and **g**. Source data

**Extended Data Fig. 5:**
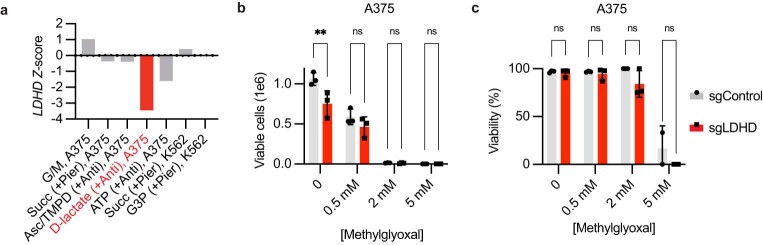
*LDHD* loss does not greatly increase sensitivity to methylglyoxal toxicity. **a**, Mean *Z* scores for *LDHD* across screens. **b**, Cell counts of control and *LDHD* KO cells in A375 after 3 days of methylglyoxal treatment at the indicated dose. **c**, Viability of control and *LDHD* KO cells in the same samples as in (**b**). Shown is the mean +/- SD, n = 3. **p = 0.0016 indicates P values for the specified comparisons from two-way ANOVA after adjustment for multiple comparisons (Tukey’s). Source data

We find that in permeabilized mammalian cells, addition of d-lactate can mildly elevate the membrane potential under antimycin conditions (Fig. 3a). By performing PMF-seq using d-lactate as the substrate and comparing it to ascorbate/TMPD (which is also known to feed into cytochrome *c*) under antimycin treatment in human A375 cells, we observed differential dependency on *LDHD* (Fig. 3b). Importantly, this dependency on *LDHD* is specific to the d-lactate screen but not seen in other substrate screens in this study (Extended Data Fig. 5a).

We proceeded to validate the screening result in A375 and HepG2 *LDHD* knockout (KO) cell lines. In these KO cell lines, we used real-time, spectrophotometric measurements of Δ*Ψ*_m_ in permeabilized cells. We found that, although they could mount a membrane potential response to ascorbate/TMPD (an artificial substrate combination that feeds directly into cytochrome *c*), their response to d-lactate was blunted (Fig. 3c). Of note, mammalian LDHD has been associated with the detoxification pathway for methylglyoxal, a highly toxic byproduct of glycolysis^23^. To explore whether LDHD is important in detoxification, we tested the wild-type and KO cells’ sensitivity to methylglyoxal but saw little difference (Extended Data Fig. 5b,c).

To firmly establish the mechanism of action of human LDHD, we expressed and purified it for in vitro biochemical characterizations. Human LDHD, which has a predicted molecular weight of ~50 kDa, ran as a dimer during gel filtration chromatography (Fig. 3d,e). Using the purified LDHD, we could confirm direct electron transfer from d-lactate to cytochrome *c* by monitoring its haem absorbance (Fig. 3f). We further characterized the steady-state enzyme kinetics and found the *k*_cat_ and *K*_M_ of this reaction to be 103 min^−1^ and 112 μM, respectively. Consistent with a recent study^24^, we identified d-2-hydroxybutyrate as another LDHD substrate that can be used to reduce cytochrome *c* (Fig. 3g). These studies provide strong evidence that human cells can modestly energize their mitochondria in an LDHD-dependent manner by directly transferring electrons from d-lactate to cytochrome *c*. Future studies will be required to determine whether the bioenergetic role of LDHD (as opposed to only the detoxification role) may be important in disease pathogenesis, or perhaps provocatively, harvesting energy from end products of bacterial metabolism.

**Fig. 4:**
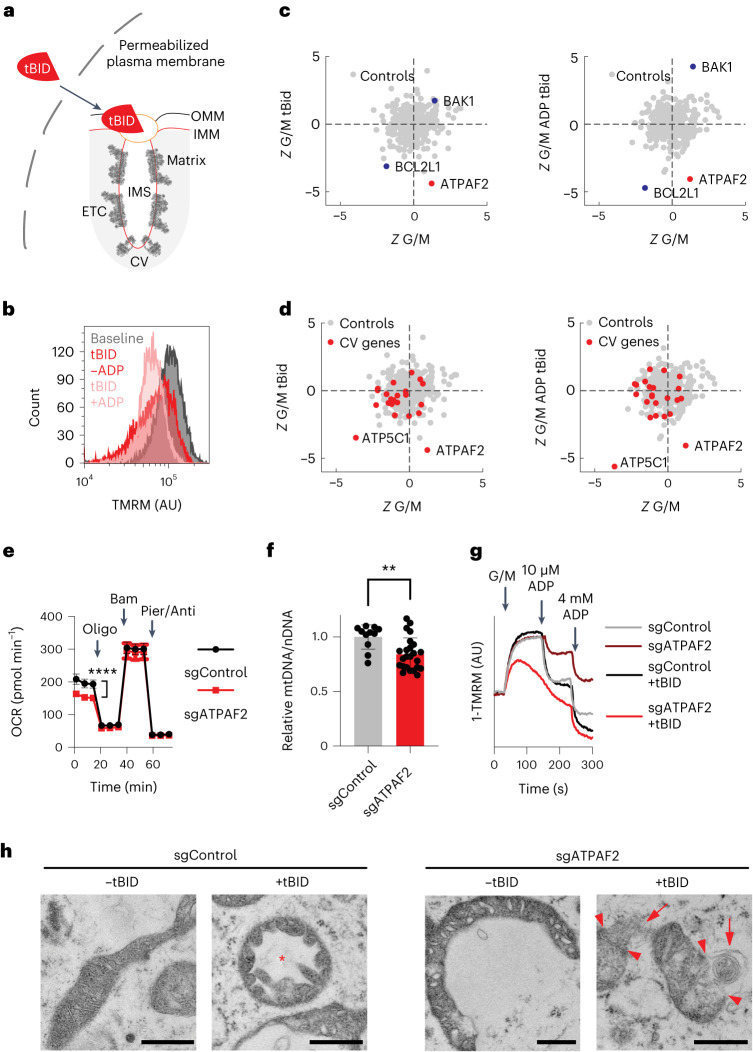
PMF-seq reveals *ATPAF2* loss as a sensitizer of acute tBID action. **a**, Schematic diagram of acute targeting of tBID to mitochondria. **b**, Endpoint Δ*Ψ*_m_ as measured by a flow cytometer in permeabilized A375 cells treated with 100 nM tBID (red) or 100 nM tBID + 4 mM ADP (light pink) compared with the G/M baseline (dark grey). Distributions of TMRM fluorescence values are shown at *t* = 10 min post treatment on flow cytometry. **c**, Scatterplots of *Z*-scores of specific hits under the tBID (left) or tBID + ADP (right) treatment. **d**, Scatterplots of Z-scores as in **c**, with all complex V structural subunits and assembly factors marked in red. **e**, Seahorse oxygen consumption rate measurements in intact cells in A375 control and *ATPAF2* KO cells. Shown are averages ± s.d. for *n* = 16 biological replicates. **f**, Real-time PCR-based measurement of mtDNA relative to nDNA in A375 control and *ATPAF2* KO cells. Average ± s.d. and individual data points are presented for *n* = 12 (control) or *n* = 16 (*ATPAF2* KO) replicates. For **e** and **f**, ****P = 2.7 × 10^−11^ or **P = 0.0018 indicates *P* values for from two-tailed Student’s *t*-test. **g**, Representative kinetics of Δ*Ψ*_m_, measured as TMRM fluorescence, in permeabilized A375 control and *ATPAF2* KO cells (±100 nM tBID). **h**, Representative transmission electron microscopy images in A375 control (left) or *ATPAF2* KO cells (right, ±100 nM tBID, +4 mM ADP). Representative mitochondria with outer membrane breakage (red arrowhead), herniation (red arrow) or highly dilated cristae with interconnected intracristal space and contracted matrix (red asterisk) are noted. Scale bars, 600 nm. For each condition in **h**, at least 15 electron micrographs were collected, with four independent images from each condition shown in Extended Data Fig. 6. ETC, electron transport chain; IMM, inner mitochondrial membrane; OMM, outer mitochondrial membrane. Source data

Finally, we sought to leverage the unique capabilities of PMF-seq to identify genetic modifiers of the death ligand tBID and specifically its acute action on mitochondria. BID belongs to the BH3-only family of pro-apoptotic proteins. During programmed cell death, activation of cell surface receptors triggers caspase-8 activation, leading to the cleavage of BID into a truncated form called tBID. This active tBID translocates from the cytosol to the outer mitochondrial membrane (Fig. 4a), where it permeabilizes the outer membrane and initiates apoptosis by prompting the release of cytochrome *c* (refs. ^25,26^). Exogenous tBID has been shown to induce a time-dependent loss of respiration in isolated mitochondria^27^. However, several aspects of tBID–mitochondria interaction remain unclear^28^. We reasoned that employing PMF-seq could allow us to identify the gene network centred on tBID-mediated acute mitochondrial damage. Crucially, the PMF-seq framework enables control of the concentration and timing of tBID addition to trigger mitochondrial damage.

We permeabilized CRISPR-mutagenized A375 cells, stained them with TMRM, energized their mitochondria using glutamate/malate and treated them with 100 nM tBID. This tBID concentration partially blunted the apparent membrane potential (Fig. 4b). Furthermore, tBID, in the presence of 4 mM ADP, further decreased the membrane potential, indicating that ADP-stimulated respiration (associated with ATP synthesis) can occur during the PMF-seq screen (within <20 min post-permeabilization). Our PMF-seq analysis using tBID as the perturbagen, compared with the baseline (glutamate/malate only), revealed that the loss of *BAK1* confers protection against membrane depolarization, while the loss of *BCL2L1* exacerbates tBID’s effects, regardless of ADP presence (Fig. 4c). The protective role of *BAK1* loss suggests that BAK probably operates downstream of tBID in our system, aligning with the fact that tBID can bind to BAK to trigger allosteric activation and intramembranous oligomerization^29^. The exacerbation of tBID’s effects by *BCL2L1* loss is consistent with prior research demonstrating that overexpressing *BCL2L1* alleviates the disruption of mitochondrial homoeostasis induced by tBID^30^. Hence, PMF-seq captures known positive and negative regulators of tBID’s acute action on mitochondria.

The complex V assembly factor gene *ATPAF2* emerged as the top aggravating hit in response to tBID treatment (Fig. 4c). This gene scored as our top hit and was not dependent on the presence or absence of ADP. Importantly, ATPAF2 stands out as the sole complex V component that exhibits essentiality under tBID treatment but not under the baseline condition (Fig. 4d). The yeast homologue of ATPAF2, ATP12, interacts specifically with the alpha subunit of complex V but is not a component of the mature complex V and, rather, is believed to guard against aggregation of alpha subunits before F_1_ assembly^31,32^. While not the focus of our subsequent investigation, the gene encoding the gamma subunit of complex V, *ATP5C1*, scores strongly even under the baseline condition, consistent with the central role of the gamma subunit rotor in dissipating the proton motive force by coupling proton translocation in F_O_ to phosphorylation of ADP in F_1._

We proceeded to characterize the *ATPAF2* KO cell line in A375 in follow-up validation studies. The loss of *ATPAF2* led only to a modest defect in baseline mitochondrial bioenergetics as evidenced by oxygen consumption rates (OCR; Fig. 4e). The KO’s OCR normalizes after treatment with the complex V inhibitor oligomycin, indicative of a reduction in ATP-coupled OCR, which is consistent with a mild complex V-related defect. Quantitative PCR (qPCR) analysis revealed a mild, 15% decrease in mitochondrial DNA copy number (Fig. 4f). Hence, loss of *ATPAF2* leads to a mild decline in mitochondrial bioenergetics and a subtle decline in mitochondrial DNA (mtDNA) copy number in intact cells.

We then conducted real-time, spectrophotometric measurements of Δ*Ψ*_m_ in permeabilized A375 control and *ATPAF2* KO cells (Fig. 4g). Although *ATPAF2* KO cells could maintain a membrane potential comparable to that of the control, they exhibited reduced depolarization in response to ADP, again consistent with complex V dysfunction. Under tBID treatment, control cells responded to ADP in a step-wise manner, indicating their continued capacity for ADP-stimulated respiration. In contrast, when *ATPAF2* KO cells were exposed to tBID, their apparent membrane potential steadily declined after addition of substrate, and they no longer responded to a low dose of ADP. Collectively, our findings demonstrate that *ATPAF2* loss, while causing only a mild defect in complex V activity on its own, potentiates tBID’s action on mitochondria, culminating in a bioenergetic catastrophe.

**Extended Data Fig. 6:**
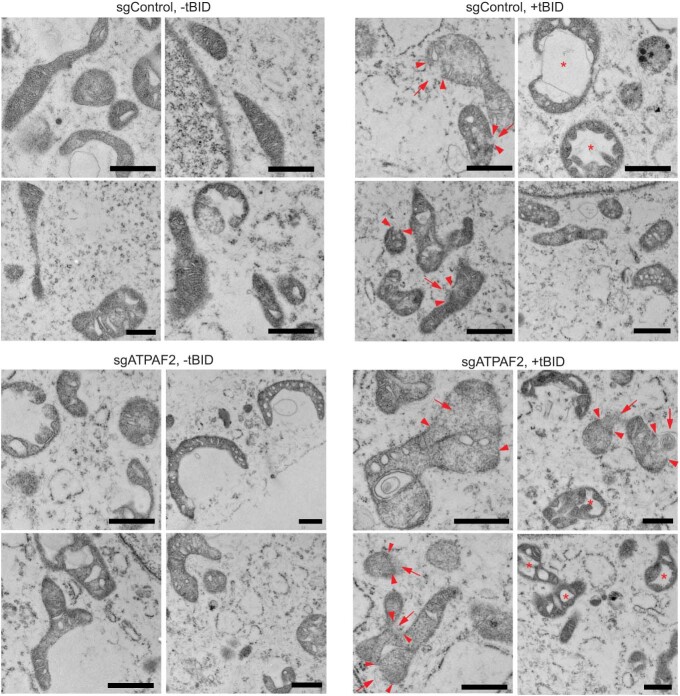
Additional data related to Fig. 4. Transmission electron microscopy (TEM) images of A375 control cells (top) and *ATPAF2* KO cells (bottom). Both control and *ATPAF2* KO cells were permeabilized and subjected to treatment with either blank buffer (-tBID) or 100 nM tBID (+tBID), as indicated. Presented here are four distinct frames of view for each condition. Representative mitochondria with outer membrane breakage (red arrowhead), herniation (red arrow), or highly dilated cristae with interconnected intracristal space and contracted matrix (red asterisk) are noted. Scale bar = 600 nm. For each condition, at least 15 electron micrographs were collected, with four independent images being displayed.

Next, we conducted transmission electron microscopy on permeabilized A375 control and *ATPAF2* KO cells to determine if the slow drift in TMRM signal and lack of response to ADP pulses in the KO cells are due to large amplitude volume changes and ultrastructural defects. Before sample collection, cells were exposed to either a vehicle control (buffer only) or 100 nM tBID for 10 min. In untreated control cells, mitochondria displayed well-defined, thin or slightly dilated, stacked, lamellar cristae with distinct crista junctions connecting to the inner boundary membrane (Fig. 4h, left). Conversely, untreated *ATPAF2* KO cells exhibited irregular inner membrane morphology, characterized by inconsistent, predominantly dilated crista shapes and infrequent crista junctions, suggesting possible detachment of cristae from the inner boundary membrane. Additionally, many mitochondria displayed elongation, pronounced curvature and a crescent-like appearance (Fig. 4h, middle right). Upon tBID challenge, control cell mitochondria underwent noticeable morphological alterations, similar to previous observations for tBID-treated liver mitochondria^33^. Cristae became irregular and swollen, and herniation and membrane breakage occurred in some mitochondria (Extended Data Fig. 6). Strikingly, some mitochondria exhibited a complete loss of normal cristae, with the inner membrane forming an expanded continuum of intracristal space that extends throughout the mitochondrion. This was accompanied by inverted inner-membrane curvature resulting in enclosure of the electron-dense matrix in ‘sausage-shaped’ compartments (Fig. 4h, middle left). Finally, tBID-treated *ATPAF2* KO cells displayed mitochondria with severe morphological derangement. Mitochondria exhibited asymmetric blebbing of herniated matrix, notable membrane rupture and swelling of both the matrix and intracristal space (Fig. 4h, right). Most notably, these mitochondria lacked any discernible cristae structure (Extended Data Fig. 6), signifying terminal catastrophe. These functional and morphological studies collectively support the notion that *ATPAF2* loss aggravates the mitochondrial actions of tBID.

Future studies are required to refine the relationship between *ATPAF2* loss and sensitivity to tBID action, but prior observations suggest possible mechanisms. First, ATP synthase is known to induce membrane curvature by forming dimer rows along cristae ridges^34^. Mitochondria in yeast mutants lacking dimer-specific subunits display severe cristae morphology changes^35,36^. *ATPAF2* loss somewhat mirrors this pattern, exhibiting crista defects (dilation and possible detachment) that worsen in the presence of tBID, which is known for its disruptive effects on cristae junctions, matrix, intracristal space^33^ and outer membrane pore formation via BAK/BAX oligomerization^26^. Second, complex V assembly intermediates might lead to formation of leak channels consisting of free c-subunits, which can induce large ion fluxes^37^. Either of these nuanced roles for deficient assembly of complex V—altered cristae morphology or formation of leak channels—could be contributing to the potentiation of tBID action. Indeed, using electron microscopy, we show that *ATPAF2* loss disrupts crista morphology, aligning with models explaining how specific complex V defects alter cristae. In fact, the crescent and ring-shaped mitochondria with large round internal spaces in the case of *ATPAF2* KO have been shown to be indicative of osmotic imbalance that can be caused by uncoupling^38^.

In this paper, we have introduced PMF-seq as a simple, high-throughput method for investigating the genetic basis of mitochondrial organelle physiology. While powerful, we point out some of its technical limitations. First, following plasma membrane permeabilization, mammalian mitochondria typically remain physiologically robust and intact for only ~30 min, which is the approximate duration of a typical FACS-based PMF-seq experiment. This limitation also affects sgRNA representation in sorted cell populations and can introduce noise especially when a weaker respiratory fuel (for example, d-lactate) is used. As such, it is crucial that FACS experiments are conducted promptly. Screening in multiple batches, and with replicates, can help to ensure high data quality. Second, both a strength and potential weakness of this approach is its sensitivity to buffer conditions. Although mitochondrial bioenergeticists experienced with classic state 3/state 4 cuvette-based types of measurements are familiar with the importance of buffer conditions, others more familiar with cell-based studies will need to optimize bulk experiments in cuvettes to ensure the mitochondria are functionally intact.

With these caveats in mind, we anticipate that the PMF-seq approach could be very broadly useful. In the current report, we focused on Δ*Ψ*_m_, the major component of the proton motive force in mitochondria. By simple extension, PMF-seq can be used to monitor other parameters classically measured in isolated mitochondria such as NADH^39^ and calcium^40^ to paint a more complete picture of mitochondrial physiology. Here we have focused on targeted CRISPR screening, but the approach can be applied to open reading frame screening and deep mutational scanning. With the goal of investigating cell death ligands acting on mitochondria, we focused on tBID, but in principle, the approach can be applied to other BH3 death agonists (BIM, BIK, NOXA, PUMA, BAX and BAK) and antagonists (BCL2 and BCL-xL) and even ‘BH3 profiling’ workflows^41,42^. Finally, we anticipate that the PMF-seq workflow can be adapted to genetic screens to investigate the physiology of other organelles such as lysosomes and chloroplasts.

## Methods

### Cell lines and cell culture

A375 (ATCC CRL-1619), K562 (ATCC CCL-243) and HepG2 (ATCC HB-8065) cells were obtained from ATCC. All experiments with wild-type cells or CRISPR–Cas9-mediated KO were performed under passage number 20 upon receipt from ATCC, and late passage cells were authenticated by STR profiling. Cells were cultured in Dulbecco’s modified Eagle medium (DMEM, Gibco no. 11995-065) supplemented with 10% foetal bovine serum (FBS, Gibco no. 26140-079) and penicillin (50 U ml^−1^)–streptomycin (50 µg ml^−1^, Gibco no. 15140-122).

### Determination of infection conditions for CRISPR pooled screens

The custom all-in-one MitoPlus library (15,271 sgRNAs targeting 1,864 genes with 8 sgRNAs per guide for most genes and 500 non-cutting control sgRNAs) was generated by the Broad Institute Genetic Perturbation Platform. The library was delivered as lentivirus. Optimal infection conditions were determined to achieve 30–50% infection efficiency, corresponding to a multiplicity of infection of ~0.5–1. Spin-infections were performed in 12-well plate format with 1.5 × 10^6^ cells in each well. Optimal conditions were determined by infecting cells with different virus volumes (0, 100, 200, 400, 600 and 800 µl) with a final concentration of 4 µg ml^−1^ polybrene in A375 cells. Cells were centrifuged for 2 h at 1,000*g* at 37 °C. Approximately 24 h after infection, cells were collected and supplemented with 1 µg ml^−1^ puromycin. Cells were counted 3 days post-selection to determine the infection efficiency, comparing survival with and without puromycin selection. Volume of virus that yielded 30–50% infection efficiency was used for screening.

### CRISPR screens with the MitoPlus ‘all-in-one’ library

Infection, selection and expansion were performed in two distinct replicates. Screening-scale infections were performed with the pre-determined volume of virus in the same 12-well format as the viral titration described above and pooled 24 h post-centrifugation. Infections were performed with 3.6 × 10^7^ cells per replicate to achieve a representation of at least 500 cells per sgRNA following puromycin selection (8 × 10^6^ surviving cells). Approximately 24 h after infection, all wells within a replicate were pooled and were split into T175 flasks, and cells were selected with puromycin for 3 days to remove uninfected cells. After selection was complete, at least 5 × 10^6^ of A375 cells were seeded in T175 flasks. The cells were passaged in fresh media (high-glucose DMEM supplemented with pyruvate, uridine and 10% regular FBS, penicillin/streptomycin) every 2–3 days. Cells were collected ~15 days after infection for PMF-seq.

### Flow cytometry-based screening with TMRM as the readout in permeabilized cells

A375-MitoPlus cells were collected in sequential batches. Each batch of cells (~200 × 10^6^) was resuspended in Agilent Seahorse XF DMEM media (supplemented with 10 mM glucose, 1 mM pyruvate and 2 mM glutamine) at 10 × 10^6^ ml^−1^ (~20 ml total). Cells were seeded in an ultralow-binding six-well plate up to 4 ml per well and were incubated at 37 °C in a CO_2_ incubator before sorting. Permeabilization buffers were prepared with the following: 1× MAS buffer (70 mM sucrose, 220 mM mannitol, 5 mM KH_2_PO_4_, 5 mM MgCl_2_, 2 mM HEPES and 1 mM EGTA), 4 mM ADP (except for the antimycin + ATP condition), 5 µM oligomycin (except for the antimycin + ATP condition), 0.7% fatty acid-free bovine serum albumin (BSA), 10 nM Seahorse plasma membrane permeabilizer (PMP, Agilent) and 100 nM TMRM (Invitrogen). At this concentration, TMRM signal behaves in the non-quenching mode where inner membrane polarization leads to a stronger TMRM signal. Right before each sort, 2 × 10^7^ cells were collected and spun down at 600*g* for 0.5 min. Media were aspirated and replaced with 1 ml of permeabilization buffer + treatment (respiratory chain substrate and small-molecule OXPHOS modulator). When cells were ready for sorting, the 2 × 10^7^ cells were transferred to a flow cytometry tube through the strainer cap. We used the following conditions in this paper: 10 mM glutamate + 10 mM malate, 1 µM piericidin + 10 mM succinate, 1 µM piericidin + 25 mM glycerol 3-phosphate, 1 µM antimycin + 10 µM ascorbate + 0.1 µM TMPD, 1 µM antimycin + 25 µM d-lactate and 1 µM antimycin + 5 mM ATP. All chemicals were purchased from Sigma-Aldrich, except for piericidin (Enzo Life Sciences) and ATP (ThermoFisher). All solutions were pH-adjusted to 7.2–7.4 with KOH.

A Sony SH800 flow cytometer was used for all cell sorting experiments in this study. Sample flow rate was adjusted to achieve an event rate of ~10,000 s^−1^. Cell population was first gated by forward versus side scatter, and the top and bottom ~15% of the TMRM distribution were sorted. Cells were sorted until all cells were consumed. For an input of 2 × 10^7^ cells, around 7.5 × 10^5^ cells could be collected from each ‘tail’. Genomic DNA isolation, PCR, sequencing and subsequent analyses of the screening results were performed as previously described^9^. For each replicate and each condition, the *Z*-score represents the *Z*-score transformation of mean log_2_ fold difference in sgRNA abundances between the low tail and high tail for each gene in each treatment.

### Analysis of the screening results

Next-generation sequencing was performed. For each condition the abundances of each sgRNA in the ‘high tail’ and ‘low tail’ were quantified and compared to identify gene KOs that are depolarized or hyperpolarized under the said condition. For a given substrate, we created scatter plots of *Z-*scores, which are derived from the mean log_2_ fold difference in sgRNA abundances between the low tail and high tail for each gene. A highly negative *Z-*score indicates the enrichment of a gene KO in the ‘low tail’ and, thus, a depolarized state, whereas a highly positive *Z*-score indicates the enrichment of a gene KO in the ‘high tail’ and, thus, a hyperpolarized state. We used a set of non-expressed genes (bottom 300 genes as determined by the abundance of transcripts) in the MitoPlus library as the controls. These controls have tight *Z*-score distributions centred around zero in all cases. To visualize the ‘electron flow’ that supports membrane potential generation under each substrate, we highlighted the gene sets for the complexes I–IV and cytochrome *c* (Fig. 2a) in two biological replicates. A gene set is required for membrane potential generation if its members are enriched in the lower left quadrant.

### Gene-specific CRISPR–Cas9 KOs

The two best sgRNAs from the MitoPlus library were ordered as complementary oligonucleotides (Integrated DNA Technologies) and cloned into pLentiCRISPRv2 (Addgene no. 52961). An sgRNA (CTTGAGACTGAGTCAGACCA) targeting a non-expressed gene OR4N4 was used as a cutting negative control. Lentiviruses were produced according to Addgene’s protocol, and cells were selected with 2 µg ml^−1^ puromycin 24 h post-infection. Puromycin was withdrawn 48 h later, and cells were maintained for 10–20 additional days during which experiments were performed. Gene disruption efficiency was verified by protein immunoblotting. The sequences of the sgRNAs used are as follows: LDHD sg1 (AGGTTCGCGAGTCCTACCCA), LDHD sg2 (CACCGCGGCAGTGGACACGT), ATPAF2 sg1 (TGTCCATTACCAGATGGTGT) and ATPAF2 sg2 (TCTTTCTTACAGAAAGGAAG). The resulting KOs were evaluated by western blotting against LDHD (Sigma-Aldrich HPA0066148) with a dilution of 1:1,000.

### Oxygen consumption measurement in permeabilized cells

For Seahorse OCR measurements with the XFe96 analyser (Agilent), A375 cells were seeded at 1.5 × 10^4^ cells per well in 80 µl per well growth media and grown overnight at 37 °C. Seahorse cartridges were hydrated overnight at 37 °C, according to the manufacturer’s protocol. After 16–20 h, cells were washed once with 1× MAS buffer (70 mM sucrose, 220 mM mannitol, 5 mM KH_2_PO_4_, 5 mM MgCl_2_, 2 mM HEPES and 1 mM EGTA). Cells were then permeabilized with 1× MAS buffer supplemented with 0.2% fatty acid-free BSA, 2 nM XF PMP (Agilent 102504-100) and 4 mM ADP. Piericidin (1 µM) or antimycin (1 µM) might be added to the buffer depending on the respiratory chain substrate under investigation. Upon assay start, baseline respiratory rate measurements were taken, followed by injection of 10 mM respiratory chain substrate, 2 µM oligomycin, 8 µM BAM15 and 1 µM antimycin (or 20 mM sodium azide when antimycin was already added). All chemicals were purchased from Sigma-Aldrich, except for piericidin (Enzo Life Sciences).

### Kinetic measurements of TMRM fluorescence in permeabilized cell

Kinetic TMRM fluorescence measurements in permeabilized cells were performed using an LS-55 fluorescence spectrometer (PerkinElmer). A total of 1 × 10^6^ A375 cells were collected and pelleted, washed once in dPBS and resuspended in 500 µl 1× MAS buffer containing 2 nM XF PMP and 0.2% fatty acid-free BSA. The 500-µl cell suspension was transferred to a quartz (Suprasil) cuvette with a 4-mm magnetic stirrer. Fluorescence was measured at an excitation wavelength of 530 nm and emission wavelength of 600 nm (with slits of 5 nm). TMRM (Invitrogen) was added at 500 nM (1.25 µl of 200 µM) and fluorescence was allowed to stabilize (~5 min). Substrate (see concentrations above) and chemical OXPHOS modulators (see concentrations above) were subsequently added. TMRM signal quenching was measured, and therefore, inner membrane polarization had an inverse relationship with TMRM signal. Depolarization was inferred from increased intensity (lower 1-TMRM) and hyperpolarization from decreased intensity (higher 1-TMRM).

### Purification and biochemical characterization of human LDHD

A Pichia codon-optimized construct containing the mitochondrial targeting sequence of DLD1 from *Komagataella phaffii*, followed by human LDHD isoform 2, a C-terminal TEV cleavage site, and a 10× histidine tag was cloned into expression vector pJGG (Biogrammatics) and then integrated into the genome of *K. phaffii* expression strain BG24 (Biogrammatics). The recombinant strain was used to inoculate 1 l cultures of BMGY media (Biogrammatics) containing 0.5 mg ml^−1^ G418 (Goldbio), which were grown at 30 °C with shaking for 24 h to an optical density around 30 (OD at 600 nm, unitless). Cells were pelleted at 4,000*g* and then frozen in liquid nitrogen. Cells were lysed with a Resch Mixer Mill MM40, and the powder was stored at −80 °C.

At the time of purification, lysed cells were resuspended in 50 mM HEPES pH 8, 300 mM NaCl, 0.1% Triton-X-100 (Sigma), 1 mM PMSF (Sigma), 1 µg ml^−1^ pepstatin (GoldBio), 1 µg ml^−1^ leupeptin (GoldBio), 1 µg ml^−1^ aprotinin (Sigma), 1 mM benzamidine (GoldBio), 0.1 mg ml^−1^ soybean trypsin inhibitor (GoldBio), 0.1 mg ml^−1^ AEBSF (GoldBio) and DNaseI (Sigma). The lysate was pelleted at 45,000*g* for 1 h and the supernatant loaded onto Talon cobalt resin (Takara). The column was washed, and the protein eluted with 25 mM HEPES pH 8.0, 300 mM NaCl and 300 mM imidazole. The protein was then run over a Superdex 200 10/300 GL column (Cytiva), concentrated to approximately 5 mg ml^−1^, and analysed by sodium dodecyl sulfate–polyacrylamide gel electrophoresis (SDS–PAGE) with Coomassie stain.

### Steady-state enzyme kinetics

The reaction buffer contained 50 mM potassium phosphate pH 8.5, 200 µM bovine heart cytochrome *c* (Sigma) and the required concentration of substrate. Purified LDHD protein (20 µg per reaction) was added to the reaction and the redox state of cytochrome *c* was monitored spectrophotometrically with a Cary 100 ultraviolet–visible spectrophotometer (Agilent) by the absorbance at 550 nm. The initial rates were determined using up to 30 s of the linear portion of the trace. Values of *k*_cat_ and *K*_M_ were determined using Prism 10 (GraphPad Software). The relative rates among various substrates were determined from the initial reaction rate of LDHD and cytochrome *c* with 10 mM substrate.

### Methylglyoxal toxicity

A375 control and *LDHD* KO cells were seeded at 5 × 10^4^ per well in a 12-well plate in regular media (high-glucose DMEM supplemented with pyruvate, uridine and 10% regular FBS, penicillin/streptomycin) and treated with 0–5 mM methylglyoxal (Sigma), followed by 3 days of growth. Cell counts and cell viability were determined by a Beckman Coulter Vi-Cell XR cell viability analyser.

### Oxygen consumption measurement in intact cells

Intact cell oxygen consumption and extracellular acidification rates (OCR and ECAR) were determined using a Seahorse XFe96 analyser (Agilent). A375 cells were seeded at 1.5 × 10^4^ cells in 100 ml per well in 96-well Seahorse cell culture plates, in DMEM supplemented with 10% FBS (Gibco no. 26140-079) and penicillin/streptomycin (Gibco no. 10378-016). After 14 h, 80 ml of media was removed and 160 ml of HEPES-buffered Seahorse DMEM (Agilent no. 103575-100) supplemented with 10 mM glucose (Sigma no. G8270), 1 mM pyruvate (Agilent 103578-100) and 2 mM glutamine (Agilent 103579-100) was added, and the plate was transferred to a 37 °C non-CO_2_ incubator for 1 h. The Seahorse cartridge was hydrated according to the manufacturer’s protocol. Oligomycin A (Sigma), BAM15 (Sigma) and piericidin A (Enzo Life Science) + antimycin A (Sigma) were prepared in Seahorse DMEM and added to the cells at final concentrations of 2 mmol l^−1^ (oligomycin), 2 mmol l^−1^ (BAM15) and 1 mmol l^−1^ + 1 mmol l^−1^ (piericidin + antimycin), respectively. Three baseline respiratory rate measurements were taken, followed by sequential injections of each inhibitor with three measurements each.

### mtDNA copy number analysis

For assessment of mtDNA copy number, cells were trypsinized and counted. A total of 2.5 × 10^6^ cells were collected in a 15 ml Falcon tube and pelleted by centrifugation at 800*g* for 3 min. The supernatant was aspirated. DNA was extracted from cells using the Qiagen Blood and Tissue DNeasy kit according to the manufacturer’s protocol (Qiagen no. 69506). DNA was quantified on a NanoDrop OneC (Thermo Scientific). Matching pairs of forward and reverse qPCR primers were combined at 10 µM concentration. The primers used in this assay were: ND1_fwd (tagcagagaccaaccgaacc), ND1_rev (atgaagaatagggcgaaggg), B2M_fwd (caggtactccaaagattcagg) and B2M_rev (gtcaacttcaatgtcggatgg). Reaction mixes were prepared by combining 30 ng DNA, 1 µl of 10 µM primer mix, 10 µl of iQ SYBR Green Supermix (Bio-Rad no. 1708880) and UltraPure dH2O (Invitrogen no. 10977-015) to 20 µl total volume. qPCR assays were run on a CFX96 Real-Time System (Bio-Rad). The qPCR parameters were: 95 °C for 3 min, 40 cycles of 95 °C for 10 s + 60 °C for 30 s + plate read, and melt curve ramp of 65 °C to 95 °C in 0.5 °C increments for 5 s at each temperature + plate read. Mean quantification cycle (Cq) values were calculated from technical triplicate wells. The ∆Cq values were calculated between mtDNA and nuclear DNA (nDNA) primer pairs, with relative mtDNA/nDNA content reported as 2^∆Cq^ mean normalized to the control.

### Transmission electron microscopy

A day before the experiment, A375 cells were seeded in 10-cm dishes at approximately 50% confluence, with 4 × 10^6^ cells per dish. On the day of the experiment, the cells were subjected to permeabilization, followed by energization using 5 mM each of glutamate and malate. They were then treated with either 100 nM tBID or buffer blank for a duration of 10 min before fixation. For permeabilization, a KCl buffer solution containing 125 mM KCl, 1 mM MgCl_2_, 20 mM HEPES–KOH at pH 7.2, 3 mM KH_2_PO_4_, 10 µM EGTA, 20 µM CaCl_2_ and 4 mM ADP was used with the Agilent XF PMP permeabilizer (0.2% fatty acid-free BSA and 10 nM Seahorse PMP). After the treatment, the permeabilization buffer was decanted and fixative was added to the plate to cover cells (2.0% glutaraldehyde in 0.1 M sodium cacodylate buffer, pH 7.4; vendor Electron Microscopy Sciences). Fixative was allowed to infiltrate 2–3 h at room temperature on a gentle rotator, then decanted. Cells were rinsed several times in 0.1 M sodium cacodylate buffer, then infiltrated 1 h in 1% osmium tetroxide at room temperature on a gentle rotator, followed by several more rinses in 0.1 M cacodylate buffer. Cells were gently scraped, with suspensions collected into centrifuge tubes, and pelleted (1,204*g* for 20 min at 4 °C).

Supernatant was removed and pellets stabilized in 2% agarose in PBS. Agarose-embedded pellets were then dehydrated through a graded series of ethanols to 100%, followed by a brief (10 min) dehydration in 100% propylene oxide. Specimens were pre-infiltrated 2–3 h in a 2:1 mix of propylene oxide:Eponate resin (Ted Pella), then infiltrated overnight in a 1:1 mix of propylene oxide:Eponate resin, both times on a gentle rocker at room temperature. The following day, specimens were placed into fresh 100% Eponate for several hours and embedded in 100% Eponate in flat moulds; resin was allowed to polymerize 24–48 h at 60 °C.

Thin (70 nm) sections were cut on a Leica EM UC7 ultramicrotome using a diamond knife (Diatome US), collected onto formvar-coated grids, stained with 2% uranyl acetate and Reynold’s lead citrate and examined in a JEOL JEM 1011 transmission electron microscope at 80 kV. The images were collected using an AMT digital camera and imaging system with proprietary image capture software (Advanced Microscopy Techniques).

### Statistics and reproducibility

Data are expressed as mean ± standard deviation (s.d.). All reported sample sizes (*n*) represent biological replicates or independent runs. No statistical methods were used to pre-determine sample sizes, but our sample sizes were similar to those reported in previous publications^8–10^ and these sample sizes were chosen to demonstrate moderate differences in commonly measured bioenergetics and physiological parameters. All attempts at replication were successful. Student’s *t*-tests were two-sided. Data distributions in the *Z*-score analysis, Student’s *t*-tests, and two-way analysis of variance were assumed to be normal but this was not formally tested beyond visual inspection. Student’s *t*-tests and two-way analysis of variance were performed with GraphPad Prism 10. The *Z*-score analysis and cumulative hypergeometric test statistics were performed with MATLAB R2021/R2022/R2023a. Flow cytometry data were plotted with FlowJo 10.

### Reporting summary

Further information on research design is available in the Nature Portfolio Reporting Summary linked to this article.

### Supplementary information

Supplementary Information Supplementary Fig. 1.

Reporting Summary

Supplementary Table 1 Numerical data and further analyses of the CRISPR screens.

### Source data

Source Data Fig. 1 Numerical data for Fig. 1.

Source Data Fig. 2 Numerical data for Fig. 2.

Source Data Fig. 3 Numerical data for Fig. 3.

Source Data Fig. 4 Numerical data for Fig. 4.

Source Data Fig. 5 Unprocessed gels for Fig. 3e.

Source Data Extended Data Fig. 1 Numerical data for Extended Data Fig. 1.

Source Data Extended Data Fig. 2 Numerical data for Extended Data Fig. 2.

Source Data Extended Data Fig. 3 Numerical data for Extended Data Fig. 3.

Source Data Extended Data Fig. 4 Numerical data for Extended Data Fig. 4.

Source Data Extended Data Fig. 5 Numerical data for Extended Data Fig. 5.

## Data Availability

All data generated or analysed in this study are provided as Source data or Supplementary Information in this paper. Results and further analyses of the CRISPR screens are available in Supplementary Table 1. The MitoCarta3.0 database is publicly available at https://www.broadinstitute.org/mitocarta/.

## Code availability

The codes for analysing the screening data are available as a public GitHub repository at https://github.com/ToTLScience/PMF-seq.
