# Supplementary material for: PMF-seq: a highly scalable screening strategy for linking genetics to mitochondrial bioenergetics
Source: Nat Metab. 2024 Feb 27;6(4):687–96. doi: 10.1038/s42255-024-00994-0 (PMC11052718; doi:10.1038/s42255-024-00994-0)
Supplement: Supplementary file 1 — Supplementary Fig. 1. [file 42255_2024_994_MOESM1_ESM.pdf]

nature metabolism

Letter https://doi.org/10.1038/s42255-024-00994-0

PMF-seq: a highly scalable screening strategy for linking genetics to mitochondrial bioenergetics

In the format provided by the authors and unedited

Supplementary Information

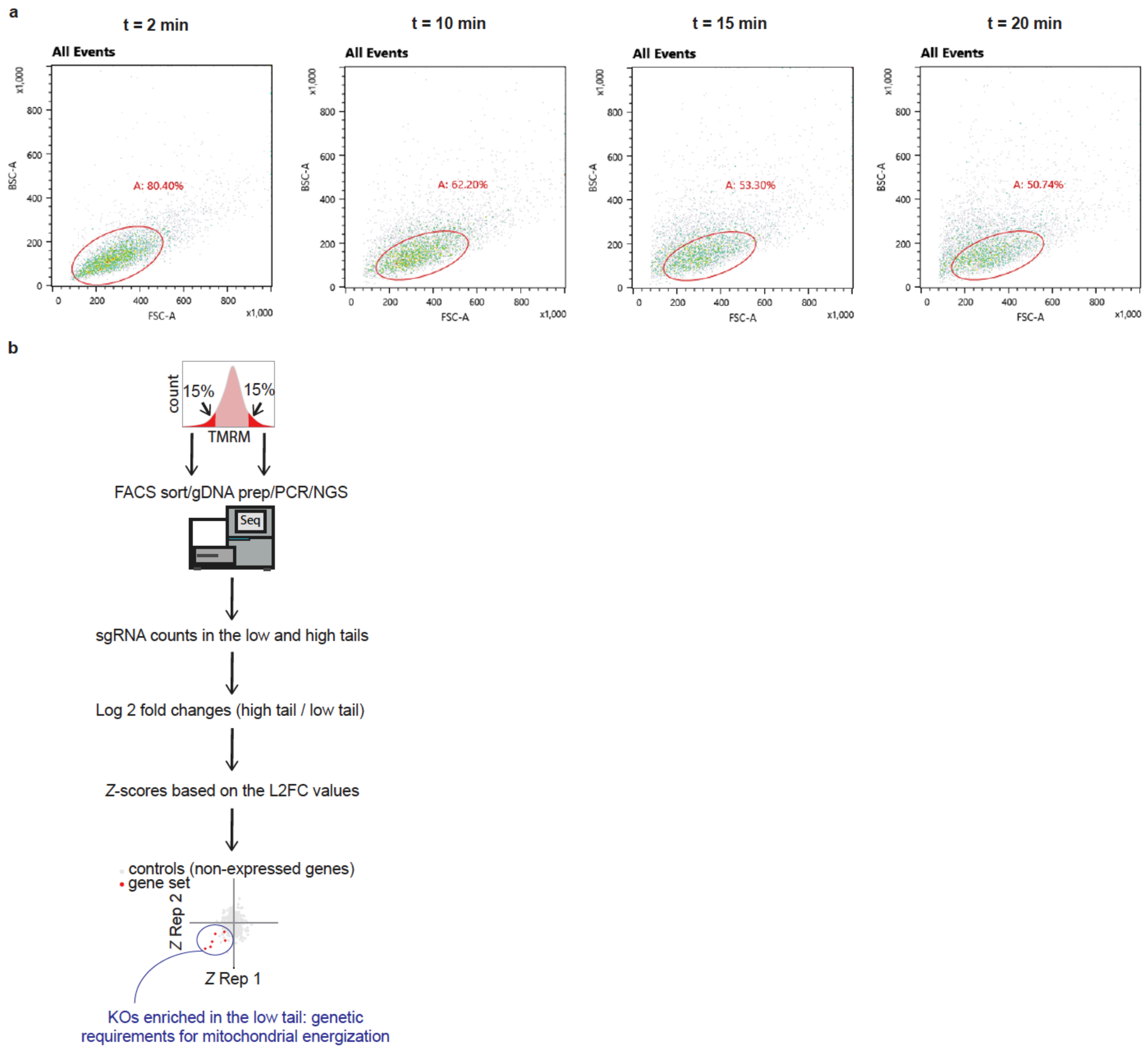

Supplementary Figure 1 | Gating strategy for flow cytometry screening and additional information on screening data analysis. a, CRISPR/Cas9 mutagenized A375 cells treated with Perfringolysis O were subjected to fluorescence activated cell sorting (FACS)**.** In each sorting experiment, the same forward versus side scatter (FSC vs. SSC) gating applies to all time points after plasma membrane permeabilization. **b**, Schematic workflow for scoring gene knockouts in FACS-based membrane potential screening.
