# Supplementary material for: PMF-seq: a highly scalable screening strategy for linking genetics to mitochondrial bioenergetics
Source: Nat Metab. 2024 Feb 27;6(4):687–96. doi: 10.1038/s42255-024-00994-0 (PMC11052718; doi:10.1038/s42255-024-00994-0)
Supplement: Supplementary file 2 — Reporting Summary [file 42255_2024_994_MOESM2_ESM.pdf]

nature portfolio

Corresponding author(s): Vamsi K. Mootha

Last updated by author(s): Jan 18 2024

Reporting Summary

Nature Portfolio wishes to improve the reproducibility of the work that we publish. This form provides structure for consistency and transparency in reporting. For further information on Nature Portfolio policies, see our Editorial Policies and the Editorial Policy Checklist.

Statistics

For all statistical analyses, confirm that the following items are present in the figure legend, table legend, main text, or Methods section.

n/a	Confirmed	
☐	☒	The exact sample size (n) for each experimental group/condition, given as a discrete number and unit of measurement
☐	☒	A statement on whether measurements were taken from distinct samples or whether the same sample was measured repeatedly
☐	☒	The statistical test(s) used AND whether they are one- or two-sided<br>*Only common tests should be described solely by name; describe more complex techniques in the Methods section.*
☒	☐	A description of all covariates tested
☐	☒	A description of any assumptions or corrections, such as tests of normality and adjustment for multiple comparisons
☐	☒	A full description of the statistical parameters including central tendency (e.g. means) or other basic estimates (e.g. regression coefficient) AND variation (e.g. standard deviation) or associated estimates of uncertainty (e.g. confidence intervals)
☐	☒	For null hypothesis testing, the test statistic (e.g. F, t, r) with confidence intervals, effect sizes, degrees of freedom and P value noted<br>*Give P values as exact values whenever suitable.*
☒	☐	For Bayesian analysis, information on the choice of priors and Markov chain Monte Carlo settings
☒	☐	For hierarchical and complex designs, identification of the appropriate level for tests and full reporting of outcomes
☒	☐	Estimates of effect sizes (e.g. Cohen's d, Pearson's r), indicating how they were calculated

Our web collection on statistics for biologists contains articles on many of the points above.

Software and code

Policy information about availability of computer code

Data collection	Illumina MiSeq Reporter was used to perform secondary analysis on the base calls during the sequence runs of amplicons. Sony SH800 software was used to collect flow cytometry data. PerkinElmer FL WinLab was used to collect fluorescence spectrophotometry data. Agilent Wave was used to collect Seahorse data.
Data analysis	A custom Python (ver. 2.7) script was used to deconvolve sequencing reads. MATLAB R2021/R2022/R2023a was used to analyze and plot screening data. FlowJo 10 was used to plot and analyze flow cytometry data. Graphpad Prism 10 was used to plot and analyze data for statistical significance.

For manuscripts utilizing custom algorithms or software that are central to the research but not yet described in published literature, software must be made available to editors and reviewers. We strongly encourage code deposition in a community repository (e.g. GitHub). See the Nature Portfolio guidelines for submitting code & software for further information.

Data

Policy information about availability of data

All manuscripts must include a data availability statement. This statement should provide the following information, where applicable:

- Accession codes, unique identifiers, or web links for publicly available datasets
- A description of any restrictions on data availability
- For clinical datasets or third party data, please ensure that the statement adheres to our policy

All data generated or analyzed in this study are provided as Source Data or Supplementary Information in this paper. Results and further analyses of the CRISPR screens are available in Supplementary Table 1. The MitoCarta3.0 database is publicly available: https://www.broadinstitute.org/mitocarta/.

Human research participants

Policy information about studies involving human research participants and Sex and Gender in Research.

Reporting on sex and gender	N/A
Population characteristics	N/A
Recruitment	N/A
Ethics oversight	N/A

Note that full information on the approval of the study protocol must also be provided in the manuscript.

Field-specific reporting

Please select the one below that is the best fit for your research. If you are not sure, read the appropriate sections before making your selection.

☒ Life sciences ☐ Behavioural & social sciences ☐ Ecological, evolutionary & environmental sciences

For a reference copy of the document with all sections, see nature.com/documents/nr-reporting-summary-flat.pdf

Life sciences study design

All studies must disclose on these points even when the disclosure is negative.

Sample size	We did not use power analysis and no statistical methods were used to predetermine sample sizes. For cell culture experiments, sample sizes were chosen to demonstrate moderate differences in commonly measured bioenergetic and physiological parameters as these are typical sample sizes reported for such experiments in the literature (e.g. PMID: 35513392, PMID: PMID: 37198474). CRISPR screens were performed in biological duplicate, a standard practice for large-scale screens (PMID: 26780180).
Data exclusions	No samples were excluded.
Replication	Experiments were repeated as detailed in the figure legends or the Method section. All attempts at replication were successful
Randomization	Randomization was not relevant in the experiments in this study which were all performed in vitro.
Blinding	Blinding was not relevant for the experiments in this study since all were performed with cultured cell lines from a commercial source.

Reporting for specific materials, systems and methods

We require information from authors about some types of materials, experimental systems and methods used in many studies. Here, indicate whether each material, system or method listed is relevant to your study. If you are not sure if a list item applies to your research, read the appropriate section before selecting a response.

Materials & experimental systems

n/a	Involved in the study	
☐	☒	Antibodies
☐	☒	Eukaryotic cell lines
☒	☐	Palaeontology and archaeology
☒	☐	Animals and other organisms
☒	☐	Clinical data
☒	☐	Dual use research of concern

Methods

n/a	Involved in the study	
☒	☐	ChIP-seq
☐	☒	Flow cytometry
☒	☐	MRI-based neuroimaging

Antibodies

Antibodies used	anti-LDHD (Sigma-Aldrich HPA0066148)
Validation	The antibody was validated by the manufacturer: https://www.sigmaaldrich.com/us/en/product/sigma/hpa066148

Eukaryotic cell lines

Policy information about cell lines and Sex and Gender in Research

Cell line source(s)	A375 (CRL-1619), K562 (CCL-243), and HepG2 (HB-8065) were obtained from ATCC.
Authentication	A375, K562, and HepG2 cells were re-authenticated by ATCC using DNA fingerprinting with small tandem repeat (STR) profilling.
Mycoplasma contamination	Cell cultures were tested for mycoplasma contamination every 3 months using Universal Mycoplasma Detection Kit from ATCC (30-1012K). All cell lines were tested negative.
Commonly misidentified lines (See ICLAC register)	No commonly misidentified cell lines were used in the study.

Flow Cytometry

Plots

Confirm that:

☒ The axis labels state the marker and fluorochrome used (e.g. CD4-FITC).

☒ The axis scales are clearly visible. Include numbers along axes only for bottom left plot of group (a 'group' is an analysis of identical markers).

☒ All plots are contour plots with outliers or pseudocolor plots.

☒ A numerical value for number of cells or percentage (with statistics) is provided.

Methodology

Sample preparation	A375 and K562 cells were treated with Agilent XF plasma membrane permeabilizer, TMRM fluorescence dye, and mitochondrial substrate and inhibitor.
Instrument	Sony SH800
Software	Collection was performed by Sony SH800 software. Data visualization and analysis were performed with FlowJo 10.
Cell population abundance	For each sorting experiment, ~2e7 cells were used as the input, and two population of ~7e5 cells each were sorted based on the TMRM fluorescence.
Gating strategy	FSC/SSC cells were used to filter out debris, dead cells, exceptionally large cells, and doublet/multiplet. Around 70% of the input cells passed this filter.

☒ Tick this box to confirm that a figure exemplifying the gating strategy is provided in the Supplementary Information.
