# Supplementary figures and images for: PMF-seq: a highly scalable screening strategy for linking genetics to mitochondrial bioenergetics
Source: Nat Metab. 2024 Feb 27;6(4):687–96. doi: 10.1038/s42255-024-00994-0 (PMC11052718; doi:10.1038/s42255-024-00994-0)

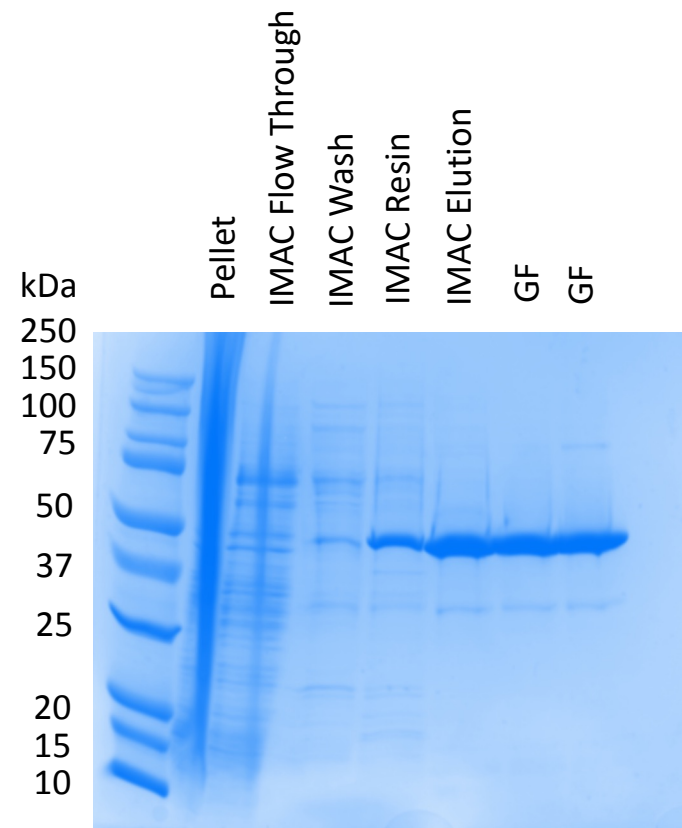

Figure 3e

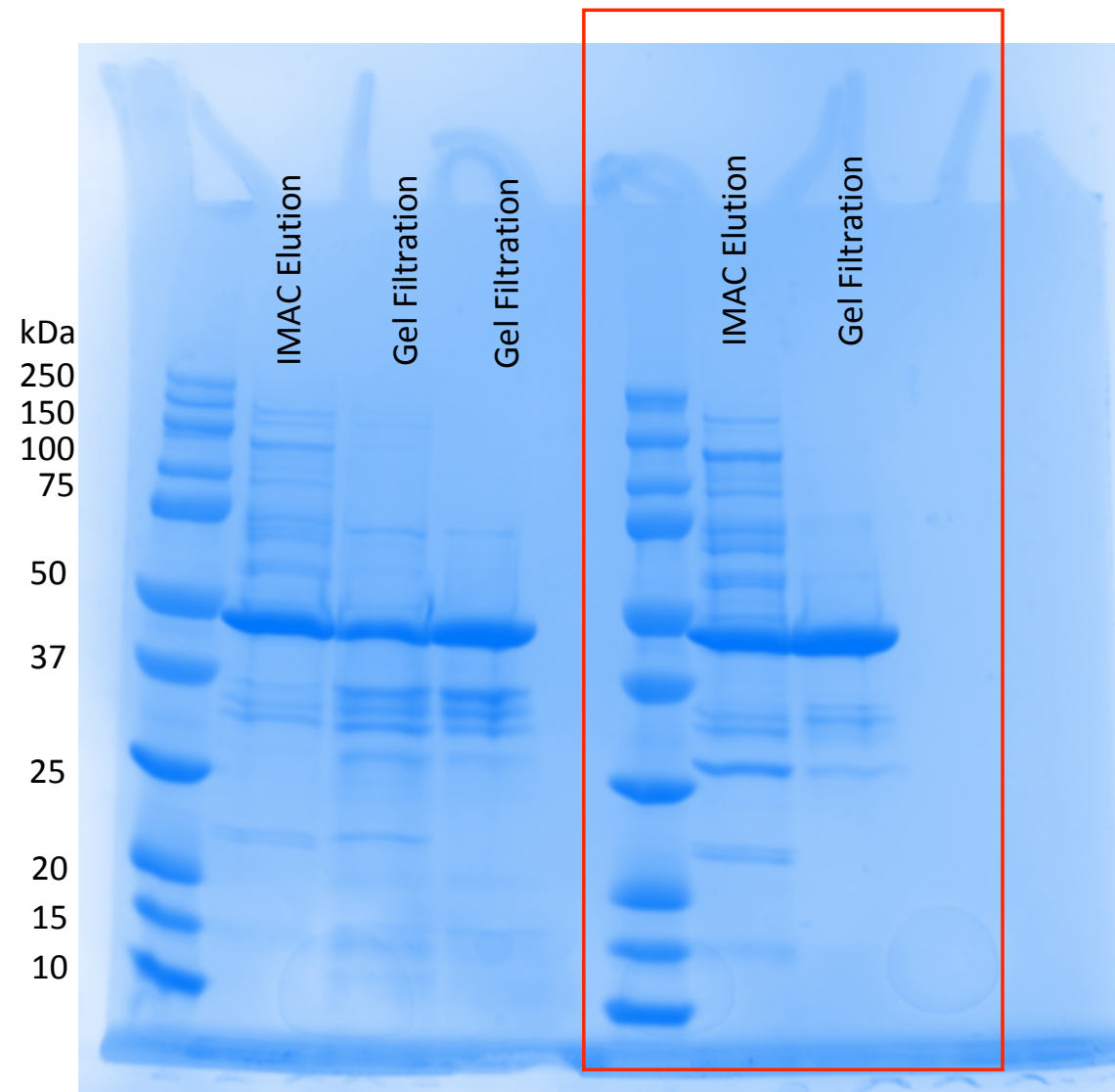

Supplement: Supplementary file 8 — Unprocessed gels for Fig. 3e. [file 42255_2024_994_MOESM8_ESM.pdf]
